# Supplementary material for: Drone-based displacement measurement of infrastructures utilizing phase information
Source: Nat Commun. 2024 Jan 9;15:395. doi: 10.1038/s41467-023-44649-2 (PMC10776656; doi:10.1038/s41467-023-44649-2)
Supplement: Supplementary file 1 — Supplementary Information [file 41467_2023_44649_MOESM1_ESM.pdf]

Supplementary Information

Drone-based displacement measurement of infrastructures utilizing phase information

Shien Ri[1]*, Jiaxing Ye[1]*, Nobuyuki Toyama[1], Norihiko Ogura[2, 3]

[1]Research Institute for Measurement and Analytical Instrumentation, National Institute of Advanced Industrial Science and Technology (AIST), Central 2, 1-1-1 Umezono, Tsukuba, Ibaraki 305-8568, Japan

[2] CORE Institute of Technology Corporation, 3-8-5 Asakusabashi, Taitou-ku, Tokyo 111-0053, Japan

[3] iTi Laboratory, Department of Civil and Earth Resources Engineering, Graduate School of Engineering, Kyoto University, Goryoohara, Nishikyo-Ku, Kyoto 615-8245, Japan

*Corresponding authors: ri-shien@aist.go.jp; jiaxing.you@aist.go.jp

Contents

Supplementary Fig. 1 Flowchart of image processing for accurate and robust deflection measurement of bridges by using a drone camera.

Supplementary Fig. 2 Marker initial angle correction and center coordinate extraction.

Supplementary Fig. 3 Principle of the sampling moiré (SM) method for accurate displacement measurement using a moiré marker with repeated patterns.

Supplementary Fig. 4 Simulated results for the image blurring compensation in 4 DoF case.

Supplementary Fig. 5 Photograph of the experimental setup to assess the influence of out-of-plane rotation of the drone camera by utilizing a 6-axis moving stage.

Supplementary Fig. 6 Experimental results of accuracy verification by rotation angle change in the out-of-plane direction.

Supplementary Fig. 7 Photograph of field experimental setup of a 110-meter-long Druck-Bund bridge.

Supplementary Fig. 8 Installation of markers for bridge deflection measurement experiment.

Supplementary Fig. 9 Photograph of large marker attachment in field experiment.

Supplementary Fig. 10 Photograph of the bridge captured by the drone camera from different viewpoint.

Supplementary Fig. 11 Intermediate results of deflection measurement of 110-meter-long Druk-Bund bridge.

Supplementary Fig. 12 Analysis results of field experiments to assess the repeatability and limitations of our developed methodology.

Supplementary Fig. 13 Captured photograph depicting the experimental scene.

Supplementary Note 1 Rectify the drone image by using similarity transformation.

Supplementary Note 2 Discussion on the possibilities and limitations of the proposed approach.

Supplementary Table 1 Comparison of the state of the art for drone-based displacement measurement method.

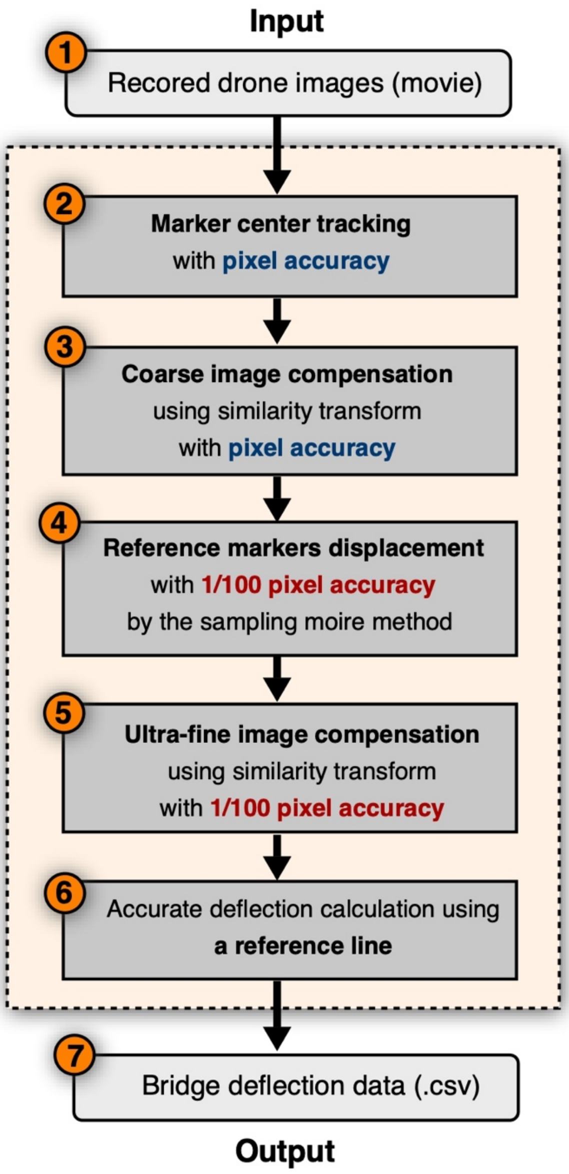

Supplementary Figure. 1 Flowchart of image processing for accurate and robust deflection measurement of bridges by using a drone camera.

The accurate and robust measurement of bridge deflection utilizing a drone camera necessitates a sequence of image processing procedures, depicted in **Supplementary Figure. 1**. The workflow includes the subsequent steps:

Step 1: Acquisition of the drone movie, which serves as the fundamental input data comprising a collection of time-series images.

Step 2: Employ marker center tracking techniques with pixel-level precision to localize the markers' positions.

Step 3: Implement the coarse compensation to image blurring by utilizing similarity transform with pixel-level accuracy to mitigate the effects of image blurring.

Step 4: Utilization of the sampling moiré method to accurately compute each marker's displacements and grating pitch with sub-pixel accuracy.

Step 5: Conducting the ultra-fine image blurring compensation utilizing similarity transform with sub-pixel marker center coordinates calculated by the sampling moiré method.

Step 6: Calculation of the final bridge deflection.

Step 7: Export the output of the bridge deflection data as a .csv file.

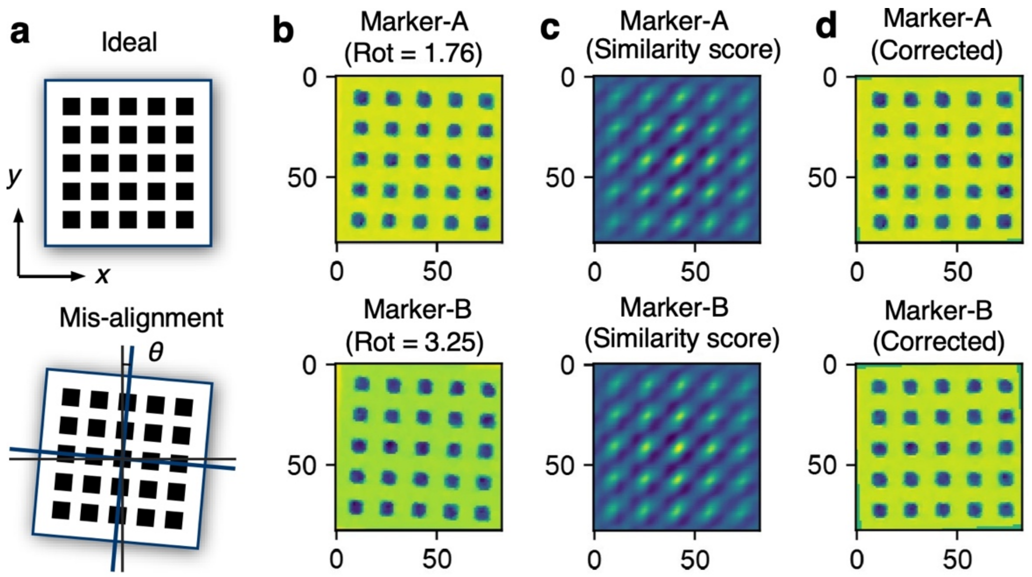

Supplementary Figure. 2 Marker's center tracking and initial angle correction. a. Ideally-fixed marker and the rotated marker with θ degree during marker attachment due to marker attachment and drone hovering position, **b.** the estimated misalignment angle based on initial angle estimation algorithm, **c.** Similarity score map for detection of marker's center coordination in pixel accuracy, and **d.** corrected marker image after angle correction to the original marker image.

Our marker center tracking process consists of two primary functions: (i) Compensation to the tilt angle of marker images which was induced by both the installation and drone camera position; (ii) Generating marker center coordinates for further displacement analysis. The results are shown in the **Supplementary Figure. 2**. We provide further explanations as follows.

First, we address the problem of the initial marker angle. Our objective is to estimate the tilt rotation angle of the marker, which is affected by two factors: **1**) The angle introduced during marker installation **2**) The relative position of the drone camera to the markers. We assume that the camera's movement with respect to the markers is negligible while hovering, making the marker tilt angle constant across all video snapshots. Based on the analysis, we manually annotate bounding boxes for all markers in the first video frame and extract their images denoted as $I_{1,\mathrm{A}}(x, y)$ with subscripts of 1 and A denote the frame and marker indexes, respectively. Let θ be the initial tilt angle to be estimated. We devised an efficient algorithm that characterizes the geometric structures of markers, such as regularity and symmetry, to achieve high-precision angle estimation.

As shown in **Supplementary Figure 2a** for an ideally-fixed marker with $\theta = 0$ degrees, the texture image $I(x, y)$ exhibits a low-rank property indicating that the marker image can be represented well by using only a few independent column-wise basis vectors. Even when a marker image encounters deformation resulting from the initial angle, the low-rank property can be restored through resembling an appropriate rotation. Based on this analysis, we introduce an objective function to estimate the local rotation of the marker. Specifically, we denote the parameter θ (in degrees) for the tilt angle and perform rank minimization using echelon forms. The process unfolds as follows:

$$\theta^* = \arg\min_{\theta}\Big(\mathrm{rank}\big(R(\theta)I(x,y)\big)\Big) \tag{2.1a}$$

$$R(\theta) = \begin{bmatrix} \cos\theta & -\sin\theta \\ \sin\theta & \cos\theta \end{bmatrix}, \quad \theta \in [-5,5] \tag{2.1b}$$

The angle correction is performed for each individual marker at the initial frame of the video, and we subsequently correct this initial angle by applying a rotation transformation in the counterclockwise direction.

Next, we discuss the marker tracking technique implemented in our system. This technique relies on the relationship between the location information of the maximum cross-correlation value and coordinate translation. This problem has been typically formulated as image registration, and we have employed an efficient The Maximum normalized Cross-Correlation (MCC) approach in our application. In the case of an aerial video composed of a series of images, extracting motion information becomes straightforward by repeatedly searching for the location of the maximum cross-correlation value between the 2D discrete Fourier transform (DFT) of the base marker image and each input object image in the subsequent frames of the video sequence. Based on the aforementioned analysis, the procedure for the maximum cross-correlation (MCC) motion extraction algorithm in the 2D frequency domain can be summarized as follows. Given a coordinate translation (x_0, y_0) and a multiplicative constant factors α, the normalized root-mean-square error between the template marker image $f(x, y)$ and input frame image $g(x, y)$ can be defined as

$$E^2 = \min_{\alpha, x_0, y_0} \frac{\sum_{x,y} | \alpha \cdot g(x-x_0, y-y_0) - f(x,y) |^2}{\sum_{x,y} | f(x,y) |^2} \tag{2.2}$$

where summations are taken over all pixel points (x, y). For a given translation (x_0, y_0), the constant factor α can be solved as

$$\alpha = \frac{\gamma_{fg}(x_0, y_0)}{\sum_{x,y} | g(x,y) |^2} \tag{2.3}$$

where

$$\begin{aligned} \gamma_{fg}(x_0, y_0) &= \sum_{x,y} f(x,y) g^*(x-x_0, y-y_0) \\ &= \sum_{x,y} F(u,v) G^*(u,v) \exp\left\{ i2\pi \left(\frac{ux_0}{M} + \frac{uy_0}{N} \right) \right\} \end{aligned} \tag{2.4}$$

in which $r_{fg}(x_0, y_0)$ is the cross-correlation of $f(x, y)$ and $g(x, y)$; the asterisk denotes complex conjugation; M and N are the template dimensions; $F(u, v)$ and $G(u, v)$ represent the two-dimensional discrete Fourier transform (2D DFT), for example,

$$F(u,v) = \sum_{x,y} \frac{f(x,y)}{\sqrt{MN}} \exp\left\{ -i2\pi \left(\frac{ux_0}{M} + \frac{uy_0}{N} \right) \right\} \tag{2.5}$$

With the above derivation, we insert the constant factor into the optimization objective:

$$E^2 = \min_{\alpha} \frac{\max_{x_0, y_0} | \gamma_{fg}(x,y) |^2}{\sum_{x,y} | f(x,y) |^2 \sum_{x,y} | g(x,y) |^2} \tag{2.6}$$

Then, the minimization problem can be resolved in the frequency domain. As a result, we record the marker center coordinates as the maximum value in the cross-correlation matrix.

Following the application of the MCC-based algorithm for marker tracking, we successfully acquired the marker center coordinates for each frame of the video. To facilitate comprehension in subsequent procedures, we represent these center coordinates as $[(c^A_{1,x}, c^A_{1,y}), \ldots (c^A_{N,x}, c^A_{N,y})]$. Let us take $c^A_{1,x}$ as an example; the superscript A indicates the marker Mk-A, and subscript 1 and x represent the first frame and x-coordinate, respectively. Through the aforementioned process, we obtain x- and y-axis coordinates for all the markers in the entire video clip. The further process of UAV camera motion compensation can be referred to **Supplementary Note 1**.

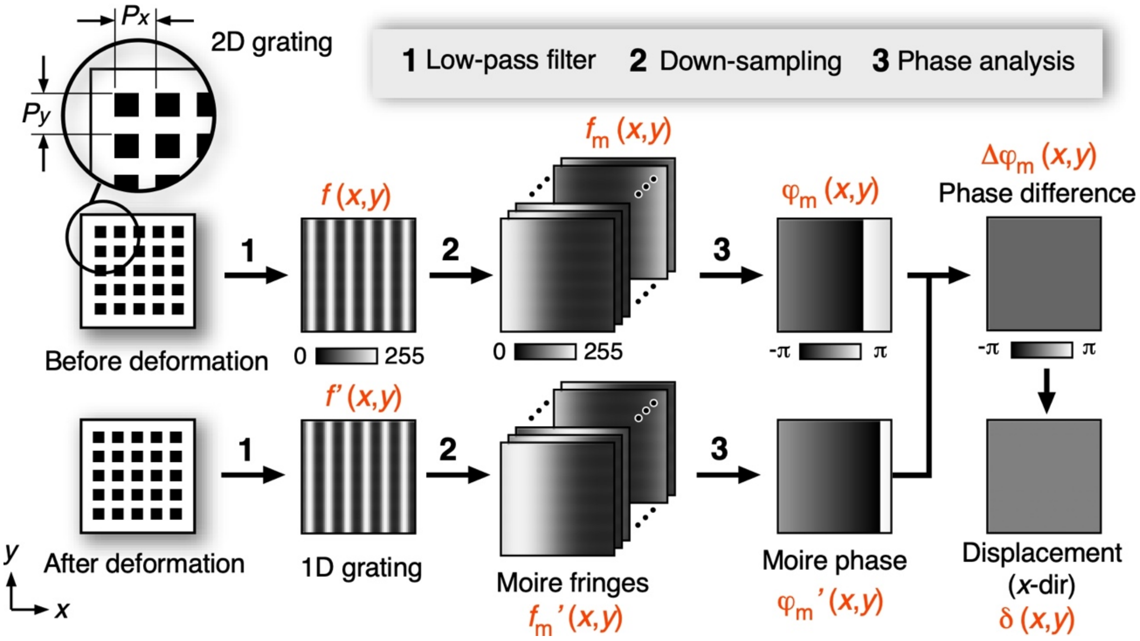

Supplementary Figure. 3 Principle of the sampling moiré (SM) method for accurate displacement measurement using a moiré marker with repeated patterns.

Supplementary Figure. 3 provides an exposition of the fundamental principle and image processing procedure employed in the sampling moiré method, thereby facilitating the accurate measurement of in-plane displacements. The essence of this approach lies in the extraction of a periodic pattern in either the *x*-direction or *y*-direction by subjecting the two-dimensional repeated pattern image, known as the moiré marker, to a low-pass filter. Remarkably, the method capitalizes on the property of moiré fringes, which serves to amplify the original repeated patterns.

In the SM method, The intensity of a 1D or 2D grating $f(x, y)$, characterized by a pitch p (in millimeters) and an initial phase φ_0, can be mathematically described by Eq. (3.1) when recorded with a digital camera.

$$\begin{aligned} f(x,y) &= a(x,y)\cos\left\{2\pi\frac{x}{P}+\varphi_0\right\}+b(x,y) \\ &= a(x,y)\cos\{\varphi(x,y)\}+b(x,y) \end{aligned} \tag{3.1}$$

where $a(x, y)$ represents the amplitude of the grating intensity, $b(x, y)$ corresponds to the background intensity, P signifies the grating pitch recorded in pixel units on the image plane, and φ represents the phase value of the grating. Since the same grating pitch in the x- and y-directions is used in typical experiments, P_x and P_y are described as P here.

Then, down-sampling using an integer sampling pitch of T-pixels and incorporating intensity interpolation enables the acquisition of multiple phase-shifted fringe patterns, denoted as $f_m(x, y; k)$, expressed as follows:

$$\begin{aligned} f_m(x,y;k) &= a(x,y)\cos\left\{2\pi\left(\frac{1}{P}-\frac{1}{T}\right)x+\varphi_0(x,y)+2\pi\frac{k}{T}\right\}+b(x,y) \\ &= a(x,y)\cos\left\{\varphi_m(x,y)+2\pi\frac{k}{T}\right\}+b(x,y), \quad (k=0,1,\cdots,T-1) \end{aligned} \tag{3.2}$$

The phase distribution of the moiré fringe, $\varphi_m(x, y)$, can be derived using the phase-shifting method with the Discrete Fourier Transform (DFT) algorithm, as represented by Equation (3.3).

$$\varphi_m(x,y) = -\tan^{-1}\frac{\sum_{k=0}^{T-1} f_m(x,y;k)\sin\left(2\pi k / T\right)}{\sum_{k=0}^{T-1} f_m(x,y;k)\cos\left(2\pi k / T\right)} \tag{3.3}$$

Similarly, the phase distribution of the moiré fringe after deformation, denoted as $\varphi'_m(x,y)$, can be evaluated using the same Equation (3.3). Subsequently, the in-plane displacement is directly derived from the phase difference distribution, represented as $\Delta\varphi_m(x,y) = \varphi'_m(x,y) - \varphi_m(x,y)$, of the moiré fringe before and after deformation. In the SM method, the zero phase point is unnecessary, as displacement can be determined from the phase difference before and after deformation.

$$\delta(x,y) = -\frac{p}{2\pi}\Delta\varphi_m(x,y) \tag{3.4}$$

Thus, to state the principle again briefly, within the sampling moiré method, multiple phase-shifted moiré fringes are acquired through the down-sampling and intensity interpolation techniques. Subsequently, the precise determination of the phase distribution of the moiré fringes is realized by employing the discrete Fourier transform (DFT) algorithm. This step of phase analysis ensures a high level of precision in the calculation process. Finally, the in-plane displacement is calculated based on the phase difference of the moiré fringe captured before and after deformation, achieving an accuracy of 1/100 pixel or 1/1000th of the grating pitch. Lastly, it is essential to emphasize that the SM method offers both accurate displacement calculations and image stabilization in our drone-based imaging technique, establishing a solid foundation that allows us to achieve an exceptional level of 1/100 pixel accuracy.

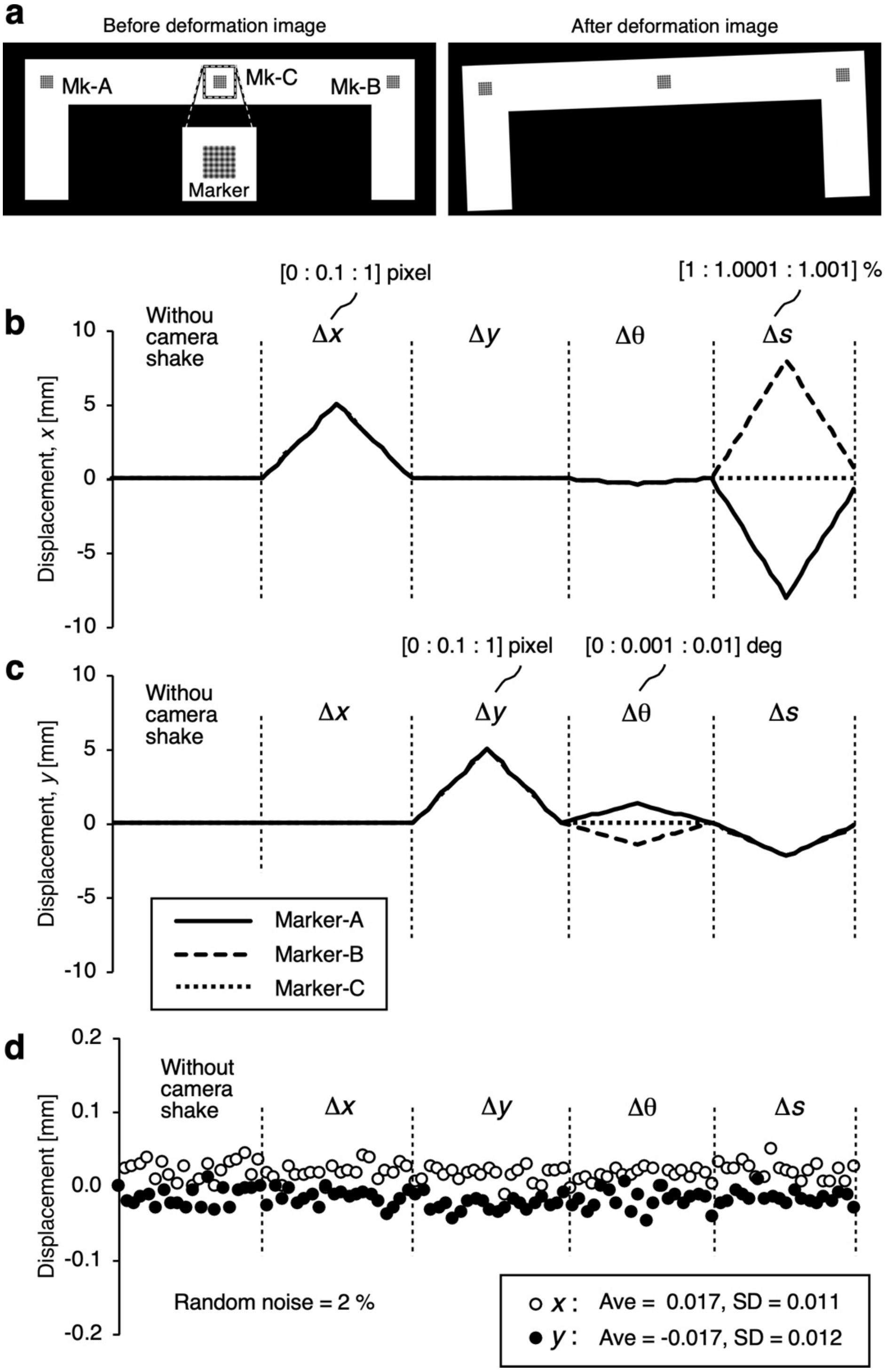

Supplementary Fig. 4 Simulated results for the image blurring compensation in the 4 DoF case. a. Simulated bridge image before deformation (left) and after deformation (right). Displacement in **b.** the *x*-direction and **c.** the *y*-direction when the image is given a translation, rotation, or scaling operation; **d.** Displacement of the center marker with image blurring compensation.

A computer simulation was performed to clarify the impact of image blurring including the in-plane rotation. A simulated bridge image, sized 4000 by 1600 pixels, underwent affine transformations involving translations in both x and y directions, rotation, and scaling to simulate the image blurring of a drone camera. Additionally, a 2% level of random noise was added to each image to account for typical camera random noise. In both *x*- and *y*-directional translations, the image underwent a series of parallel shifts ranging from 0 pixels to a 1-pixel gap, with increments and decrements of 0.1 pixels, ultimately returning to its initial position. Regarding image

rotation, a clockwise rotation of 0.001 degrees to 0.01 degrees was executed, followed by a counterclockwise rotation of 0.01 degrees, ultimately restoring the image to its original orientation; In the process of image magnification, it underwent scaling operations, initially enlarging from 1x to 1.001x at 1.0001x step, followed by reduction at 1.0001x step, ultimately restoring it to the original size of 1x. The virtual grid pitch of the markers was defined as 100 mm, and the image was analyzed with a sampling pitch of 20 pixels. Following this, the average value within a 30x30 pixel region centered on the central marker was computed.

The analysis results in **Supplementary Figure 4d** reveal that no significant disparity exists in the displacement magnitude between the scenario devoid of camera shake and the scenario where image correction for translation, rotation, and magnification is applied. This simulation confirms that the proposed method, leveraging two reference markers as “a reference line”, achieves measurement precision akin to the conventional method. Additionally, these simulation outcomes illustrate the capability of the proposed approach to rectify in-plane rotation with an accuracy of 1/100 pixel relative to the sampling moiré method, remaining unaffected by in-plane rotation.

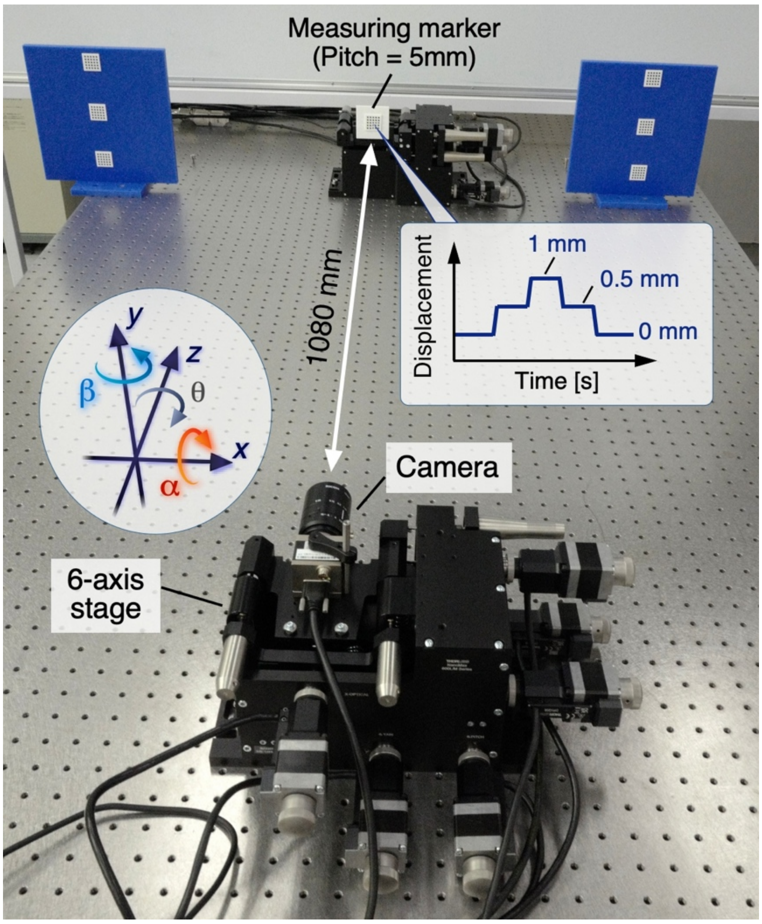

Supplementary Fig. 5 Photograph of the experimental setup to assess the influence of out-of-plane rotation of the drone camera by utilizing a 6-axis moving stage.

Supplementary Figure 5 depicts the photograph of the experimental setup. Here, the camera (Basler, acA4096-40um; monochrome color) with a focal length of 12 mm lens was fixed on a 6-axis moving stage (Thorlabs, NanoMax 6-Axis Flexure Stage MAX681/M; The theoretical resolution of X, Y, and Z are 1.8 nm, 1.8 nm, and 1.2 nm. Theoretical resolution of Roll, Pitch, and Yaw are 0.021 μRad, 0.021 μRad, and 0.021 μRad) and the rotation of the moving stage was controlled to simulate the out-of-plane rotation of the drone while hovering. In this verification experiment, the distance from the camera to the marker was 1080 mm, and from Mk-A to Mk-B was 1000 mm. The Mk-C marker was fixed to a 6-axis moving stage (Thorlabs, NanoMax 6-Axis Flexure Stage MAX681/M), and the bridge deflection measurement was reproduced by controlling the amount of movement in the *y*-direction.

Under the specified experimental conditions, the measurement marker underwent a sequence of movements, including a displacement of 0.5 mm in the *y*-direction, which was maintained for 2 seconds, followed by a further displacement of 1 mm, also held for 2 seconds. Subsequently, the marker was returned to a 0.5 mm displacement for another 2 seconds before being restored to its original zero displacement position. Concurrently, the camera's tilt angle α was continuously adjusted from 0° to 0.1°, synchronized with the onset of the measurement marker's movement. Similarly, other experiments were conducted involving continuous control of the camera's tilt angle β from 0 to 0.1 degrees, and additional experiments in which both α and β were simultaneously adjusted from 0 to 0.1 degrees.

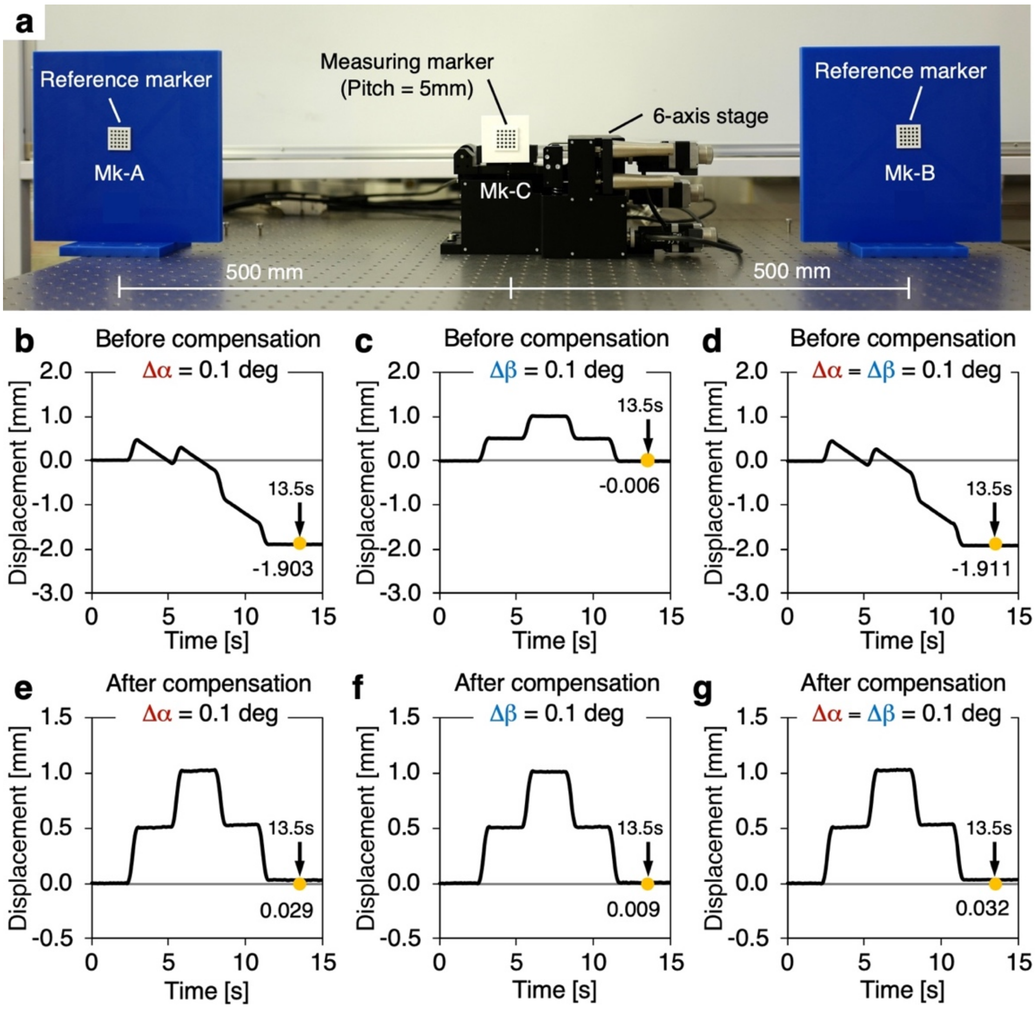

Supplementary Fig. 6 Experimental results of accuracy verification by rotation angle change in the out-of-plane direction. a Photograph of the optical system and three moiré markers with a 5 mm pitch was used. The measuring marker (Mk-C) was attached onto an accurate 6-axis moving stage. The distance between the Mk-A and Mk-C, Mk-B and Mk-C was both 500 mm. Before compensation results in the case of **b** Δα = 0.1 deg, **c** Δβ = 0.1 deg, and **d** Δα = Δβ = 0.1 deg, respectively. After compensation results in the case of **e** Δα = 0.1 deg, **f** Δβ = 0.1 deg, and **g** Δα = Δβ = 0.1 deg, respectively.

Supplementary Fig. 6 indicates the experimental results of accuracy verification by rotation angle change in the out-of-plane direction. As shown in **Supplementary Figs. 6b** and **6d**, the error is more than 1.9 mm without correction, whereas after correction, the error is reduced to about 0.03 mm. When the tilt angles of α and β, the rotation angles of the x and y axes, are 0.1 degrees, the rotation of the x-axis is slightly uncorrected, and an error of about 0.03 mm remains, as shown in Supplementary **Figs. 6e** and **6g**.

For a drone equipped with a gimbal (3-axis stabilizer), the angular motion change range, as specified in the product documentation, is less than ±0.005°. This maximum range is one-tenth of that employed in this verification experiment, resulting in a deflection measurement error due to angle changes that are reduced by a factor of one-tenth. This suggests that the impact of out-of-plane rotation on measurement accuracy is negligible. Besides, the angular change in β affects the displacement in the *x*-direction. It is incredibly insensitive to deflection measurement, which is displacement in the *y*-direction. As shown in **Supplementary Fig. 6c**, it can be confirmed that even a minute angular change in β does not affect displacement in the *y*-direction.

This verification experiment confirmed the ability of the proposed method to achieve the sub-millimeter level of accuracy necessary in the field for deflection measurements, while effectively mitigating measurement errors stemming from out-of-plane rotations during drone video capture in hovering mode.

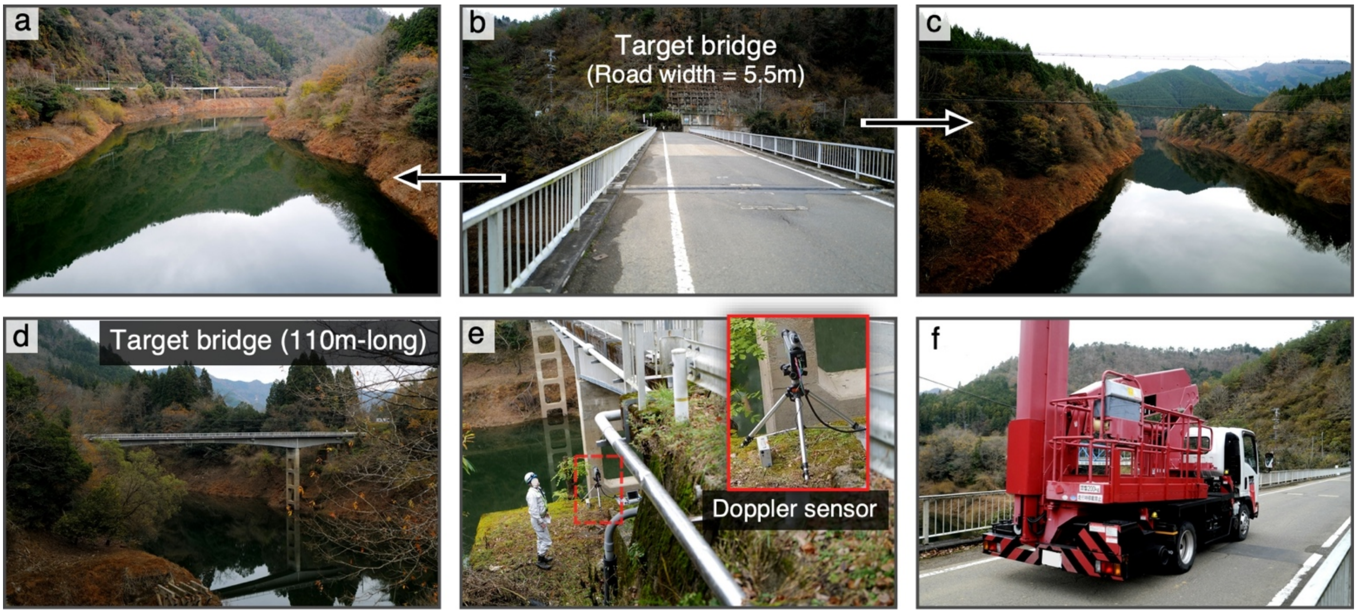

Supplementary Figure. 7 Photograph of field experimental setup of a 110-meter-long Druck-Bund bridge. **a-d** experimental scene of the target bridge, **e** location of the Doppler sensor, **f** the 8 t inspection vehicle.

The bridge that is the subject of this study is a Druck-Bund bridge, with a length of 110 m (see **Supplementary Figure. 7d**) and a road width of 5.5 m (see **Supplementary Figure. 7b**). As depicted in **Supplementary Figures. 7a** and **7c**, it was not feasible to install cameras in front of the bridge due its geological layout. However, **Supplementary Figure. 7e** illustrates a location next to the bridge girder where a conventional Doppler sensor was placed to generate reference displacement measurement. Note that such an installation location is barely available in real scenarios. A magnified view of the inspection vehicle utilized in the experiment is presented in **Supplementary Figure. 7f**. During the experiment, the vehicle, weighing 8 t, traversed the bridge at a 20 km/h velocity.

The positioning of the drone camera is linked to the measured bridge length. The proposed method requires simultaneous capturing of images of the reference markers located on the fixed girders at both ends of the bridge, in conjunction with the measurement marker positioned at the bridge's central location. Therefore, it is essential to employ a drone camera that can capture the entire bridge in a single shot. Due to the wide-angle lens of the drone camera, the shooting distance of the drone and the length of the bridge to be measured are approximately equal.

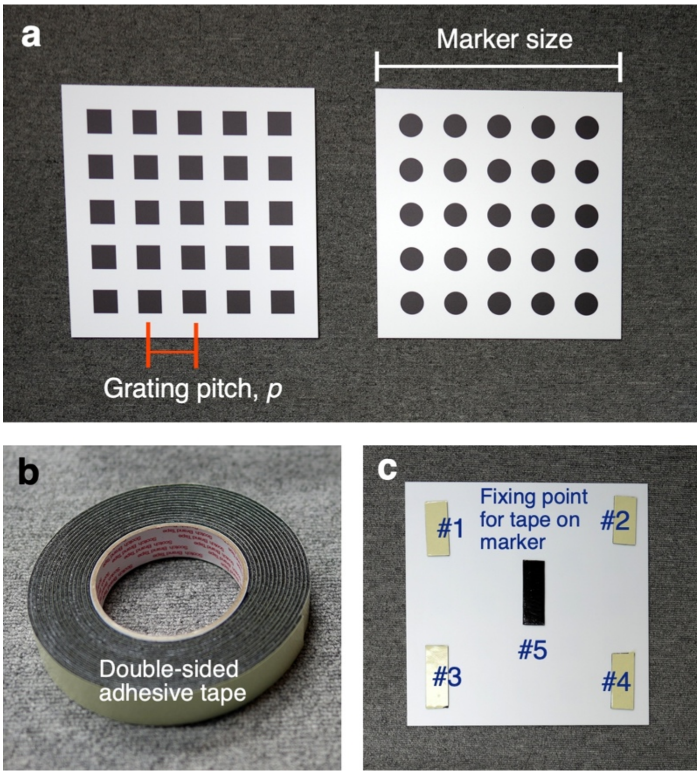

Supplementary Figure. 8 Installation of markers for bridge deflection measurement experiment. a photograph of markers designed and fabricated, **b** photograph of double-sided adhesive tape for attaching to concrete bridge and **c** fixing point for tape on the back of the marker.

Supplementary Fig. 8a shows a photograph of markers designed and fabricated in experiment. The pattern can be either square or circular, as long as the pitch remains regular. Considering the sampling moiré method's capability to detect minute displacements with a precision of 1/1000 of the grid pitch, the marker's grid pitch is determined by the desired accuracy of the measurement target. The subsequent task involves marker size determination, accounting for the camera lens specifications and shooting distance during marker capture by a drone camera. In the marker images captured in this study, each grating pitch was configured to be more than 10 pixels.

Supplementary Fig. 8b and **Fig. 8c** shows the photograph of double-sided adhesive tape for attaching to concrete bridge and the fixing point for tape on the back of the marker. The marker can be securely affixed using adhesive tape at five positions: the four corners and the center of its rear surface. This attachment not only ensures marker stability but also eases its subsequent removal.

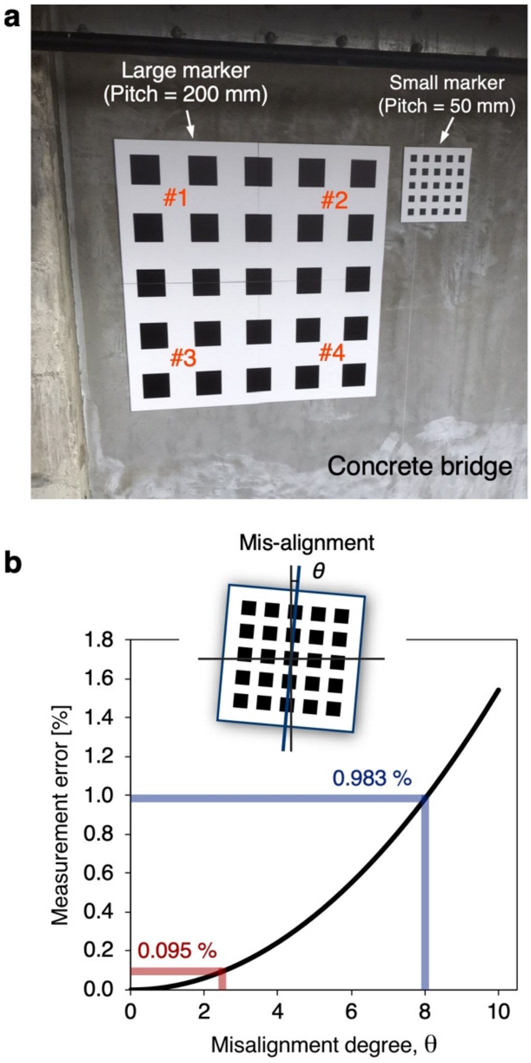

Supplementary Figure. 9 Photograph of large marker attachment in field experiment. a To enhance portability, four individual markers with a 200 mm pitch, each measuring 0.5 square meters, are merged. For comparison, a smaller marker employed in measuring the deflection of a 35-meter-long bridge is presented on the right. **b** The relationship between the misalignment angle and measurement error in percent.

In an experiment measuring the 110-meter bridge's deflection, a photograph as shown in **Supplementary Fig. 9a** depicts the site following the attachment of large markers, organized in sets of four for convenience. For comparison, a smaller marker employed in measuring the deflection of a 35-meter-long bridge is presented on the right.

The measurement accuracy is influenced by rotational misalignment when attaching the marker to the concrete bridge surface. As shown in **Supplementary Fig. 9b**, the theoretical error ratio for measured displacement is expressed as $1/\cos\theta-1$, accounting for the change in grating pitch from P to $P/\cos\theta$ due to a rotation angle θ. With a 2.5-degree deviation in the rotation angle, the error rate remains at a mere 0.1%. Although an 8-degree rotation deviation yields a 1% measurement error, as depicted in **Supplementary Fig. 2**, marker rotation during detection mitigates the angular error.

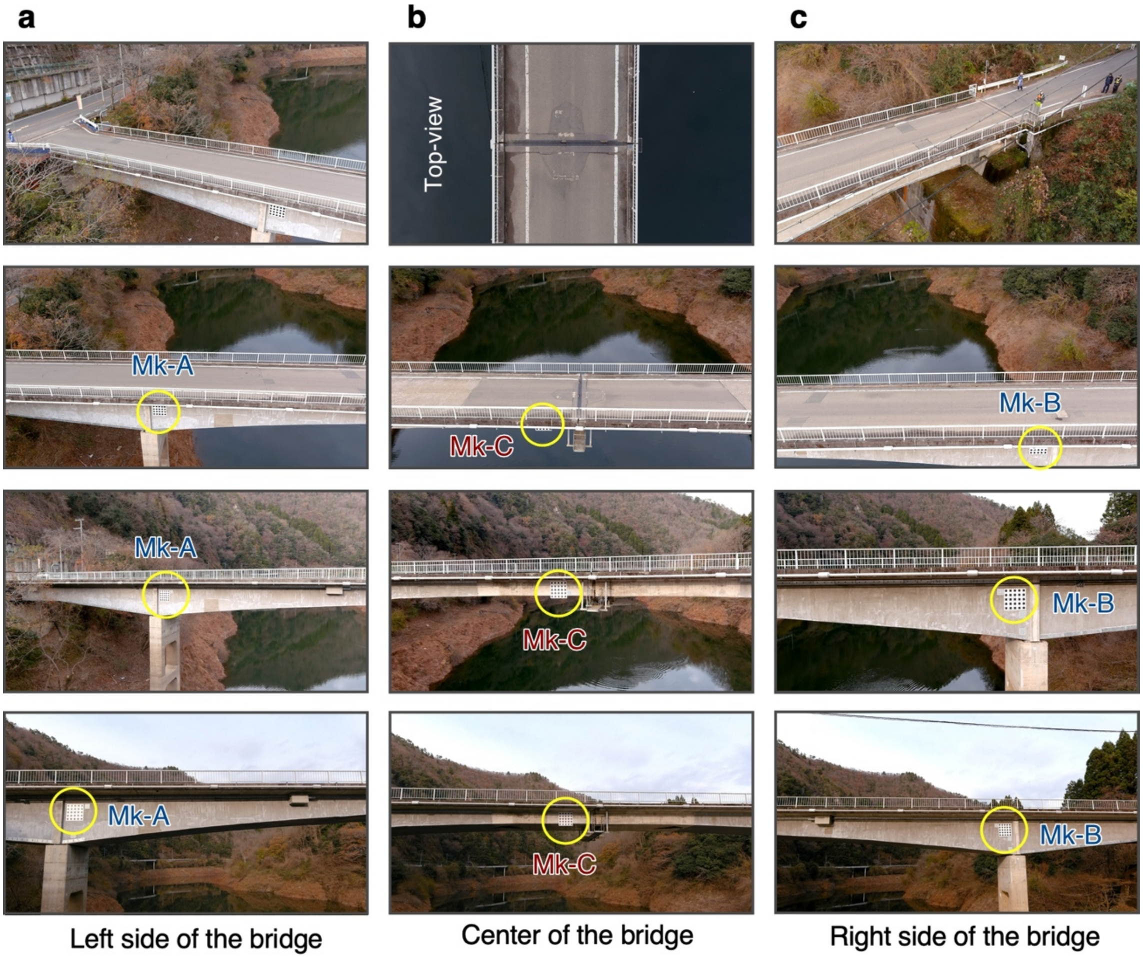

Supplementary Figure. 10 Photograph of the bridge captured by the drone camera from different viewpoint. a the left side, **b** center and **c** right side of each marker.

To give an idea of how the field experiment was conducted, i.e., the photographs of the bridge and markers captured by a drone, we present the details in **Supplementary Figure. 10**. Each marker with a pitch of 200 mm is attached to the bridge's left, center, and right girders, respectively, with double-sided adhesive tape (3M sign & display structural joint tape, T410; tape thickness is 1.0 mm). In this experiment, the markers were applied in advance of aerial photography capture using an elevated work vehicle (as shown in **Supplementary Figure. 10f**). Based on the findings from **Supplementary Figure 9b**, it had been validated that when the misalignment is 2.5 degrees, the measurement error remains below 0.1%. Hence, if the alignment angle for marker placement adheres to the ±2.5-degree requirement, significant issues in measurement can be avoided. Moreover, we have developed an effective algorithm, as illustrated in **Supplementary Figure 2**, to cope with the initial angle issues that may arise during marker installation. Based on the above analysis and process, it can be stated that the concerns associated with the rotation angle of marker placement could be effectively alleviated. For secure attachment of the double-sided tape onto the bridge concrete girder, it is advisable to lightly clean the girder surface by wiping off any dust and dirt with a towel. While increasing the number of reference markers can enhance the stability of deflection measurements, it should be noted that this approach necessitates additional labor for marker installation. Therefore, as a minimum requirement, two reference markers should be installed at each end of the bridge.

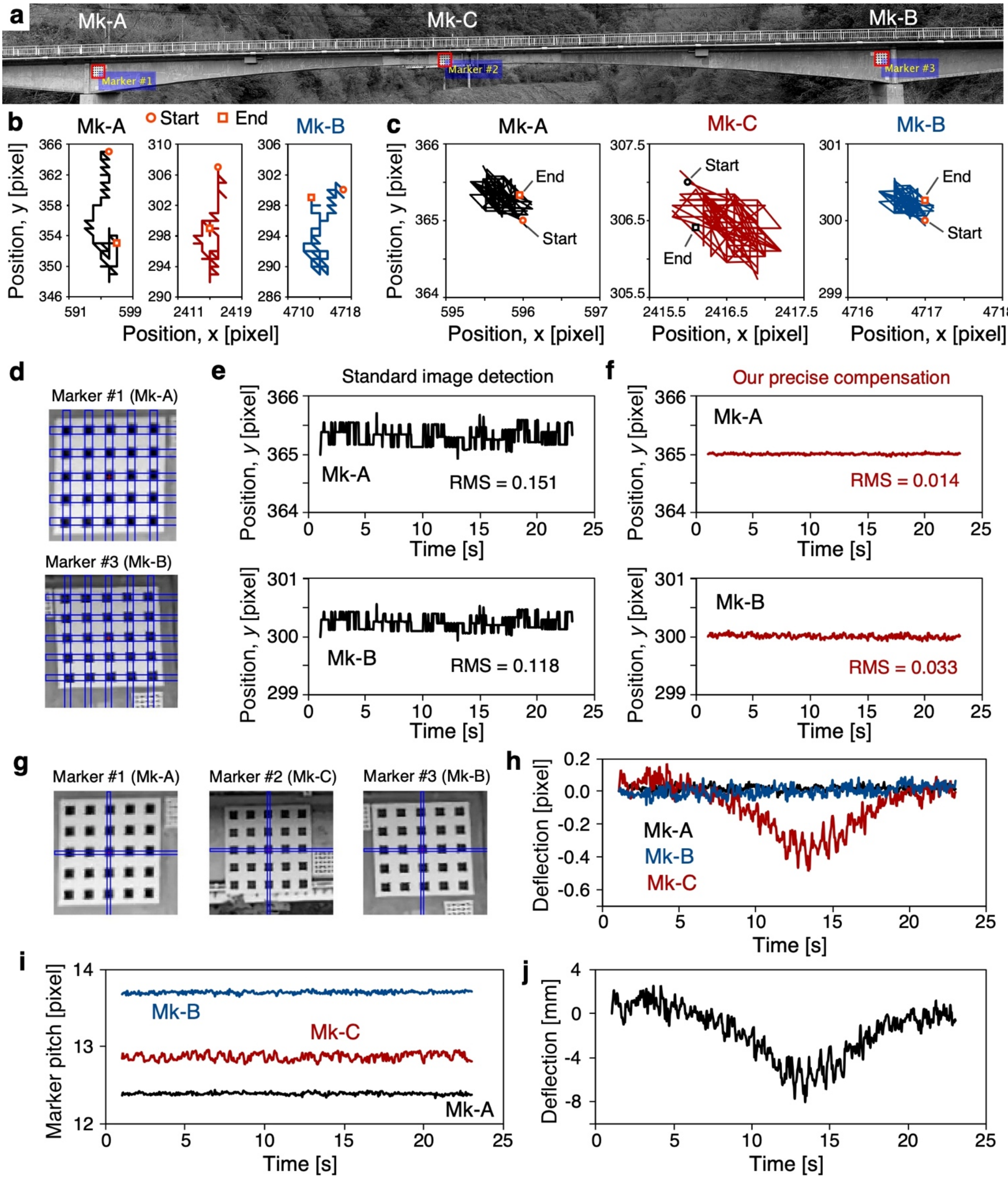

Supplementary Figure. 11 Intermediate results of deflection measurement of 110-meter-long Druk-Bund bridge. a The target bridge and the auto-detected three moiré markers. **b** The trajectory of marker center coordinates with pixel accuracy. **c** The trajectory of marker centers with sub-pixel accuracy generated by first-round-rough image stabilization (aligning the snapshot image to the initial frame by using similarity transform). **d** Marker centers detection for two reference markers (Mk-A & Mk-B). The measured *y*-directional displacement for the two reference markers after the image stabilization using in similarity transform with **e** standard image detection and **f** the refined marker center coordinates with sub-pixel accuracy by our precise compensation method, respectively. **g** Automatic detection of three marker centers. **h** The measured *y*-directional displacement of thee markers (two reference markers A and B, one measuring marker C) calculated following the second round of precise image stabilization. **i** The grating pitch of three markers in time series obtained by the sampling moiré method. **j** The final displacement of the bridge (i.e., Mk-C).

Supplementary Figure. 11a illustrates the bridge selected for measurement in this study. Three moiré markers were strategically positioned on the bridge's left and right girders, as well as at its center. Employing robust visual object tracking algorithm, we extracted the trajectory of these markers' center coordinates, as exemplified in **Supplementary Figure. 11b**. To achieve preliminary image stabilization, the center coordinates of the two reference markers (Marker #1 and Marker #3) were acquired, and we align the sequential snapshots to the initial frame of the ariel photography video by using similarity transformation. As shown in **Supplementary Figure. 11c**, it is noteworthy that the initial image stabilization successfully compensates displacements within a range of 1 pixel in both the x and y directions. It is important to mention that the displacement of the center marker is slightly magnified due to the deflection induced by the passage of the test vehicle.

Supplementary Figure. 11d depicts the marker centers detection for two reference markers (Mk-A & Mk-B). The measured y-directional displacement for the two reference markers after the image stabilization using in similarity transform with standard image detection and the refined marker center coordinates with sub-pixel accuracy by our precise compensation method are shown in **Supplementary Figure. 11e** and **11f**, respectively. Our method dramatically improved the RMS of the position change in the y-direction. The incorporation of the sampling moiré method enables the attainment of image stabilization with heightened precision.

Supplementary Figure. 11h and **11i** present the y-direction displacements and marker pitch of the center of three markers (See **Supplementary Figure. 11g**), calculated following the second round of precise image stabilization. Remarkably, despite using a drone for image capture, the displacement measurement exhibited exceptional stability. Subsequently, utilizing the ABC method, the relative displacements of the measurement marker (Mk-C) concerning the reference markers (Mk-A and Mk-B) were further determined, revealing the deflection as depicted in **Supplementary Figure. 11j**. Furthermore, we employed a smoothed moving average (SMA) filtering procedure with a filter size of four pixels to mitigate camera noise further, thereby yielding the final deflection outcome as indicated in **Figure. 4e**.

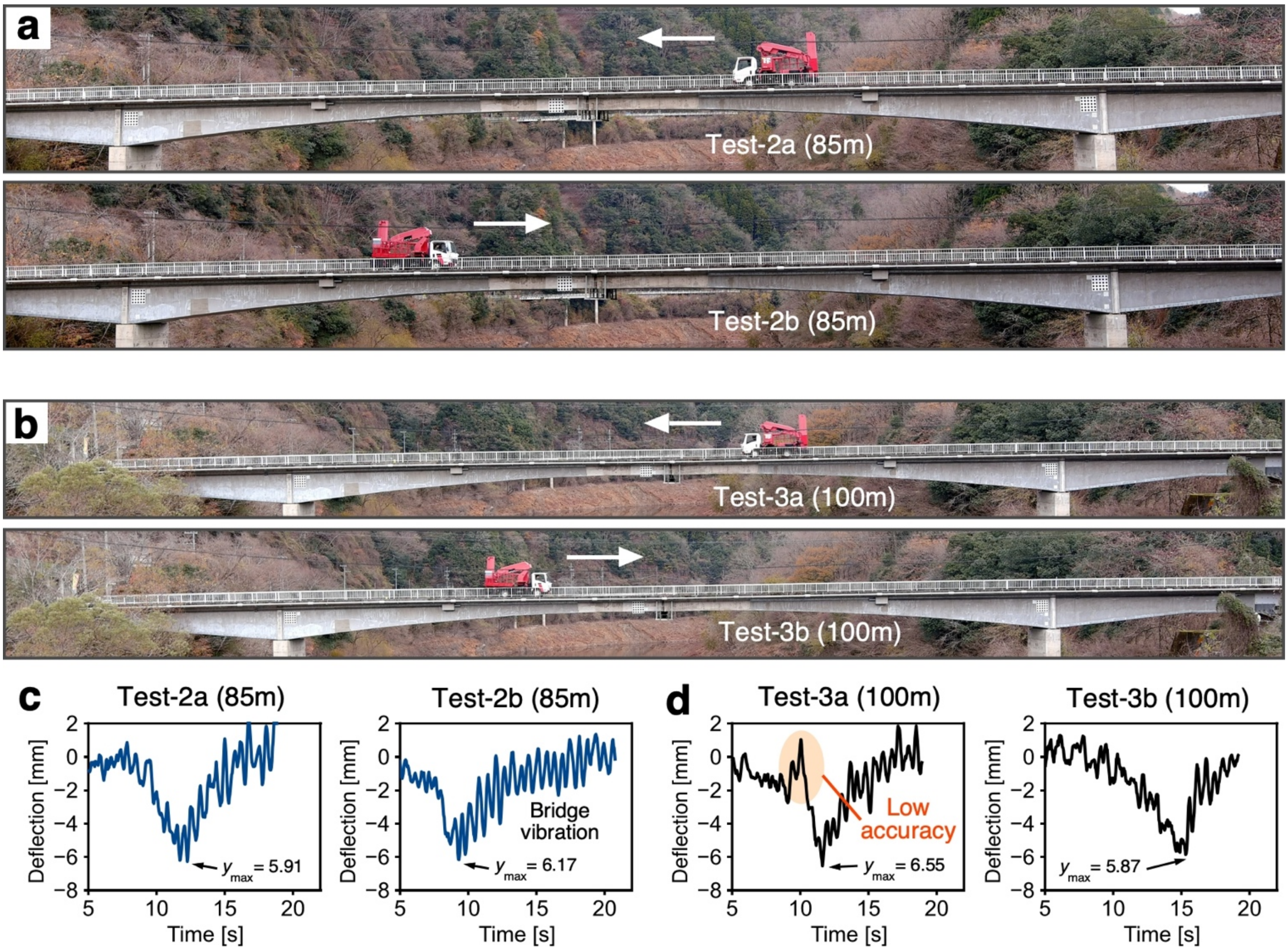

Supplementary Figure. 12 Analysis results of field experiments to assess the repeatability and limitations of the development methodology. Images recorded by drone camera at **a** 85m (test-2a and test-2b) and **b** 100m (test-3a and test-3b) shooting distance. Measured deflection at **c** 85 mm (test-2a and test-2b) and **d** 100m (test-3a and test-3b) shooting conditions.

Further deflection measurements were conducted at a distance of 85 m to assess the ***repeatability***. As depicted in **Supplementary Figure 12c**, both travel directions of the vehicle (from A1 to A2 and A2 to A1) yielded good deflection results, and similar to **Figure 4e**. In this case, the grid pitch of the markers ranged from 12.3 to 13.6 pixels. As a comparison, two deflection measurements were performed at a distance of 100 m to investigate the ***limitations*** of the proposed method. In this case, the grid pitch of the markers ranged from 9.9 to 10.1 pixels. At an imaging distance of 100 m, as depicted in **Supplementary Figure 12d**, the maximum deflection value approached 85 m. However, the time series analysis results, specifically the Test-3a outcome, revealed notable fluctuations. This observation underscores the difficulties in achieving stable deflection measurements using a drone camera beyond the 100 m imaging distance. Furthermore, in line with established imaging measurement techniques, drone imaging within a range of 85 m is deemed appropriate since the measurements are susceptible to weather-related factors such as air fluctuation and shimmer for long-distance image shooting.

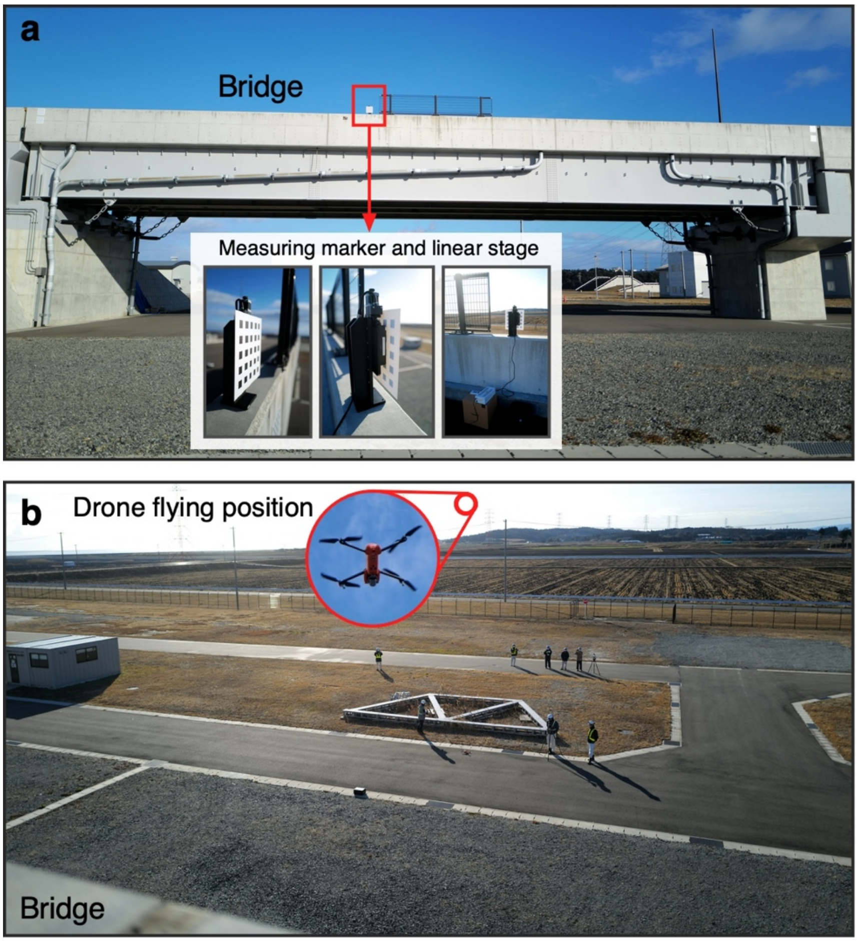

Supplementary Figure. 13 Captured photograph depicting the experimental scene. a The experimental setup for conducting extensive verification involved a 35-meter-long bridge located in Fukushima. A measuring marker was positioned near the center of the bridge and firmly affixed to a precise linear moving stage to control the displacements in the *y*-direction accurately. **b** The drone's flight position during the deflection measurement process.

The photograph of accuracy verification experiment conducted at the Fukushima robot test field is shown in **Supplementary Figure. 13**. The measurement marker fixed to a moving stage (**Supplementary Figure. 13a**) was moved up and down to simulate the occurrence of bridge deflection. Next, the flight position of the drone aerial photography in this experiment and its experimental scene is shown in **Supplementary Figure. 13b**. The drone flew to approximately the same height as the bridge and captured the video while the moving stage was in motion during a hovering state.

Supplementary Note 1: Rectify the drone images by using similarity transformation

In this section, we present the compensation model designed to address the displacements caused by camera motion. The primary objective is to achieve a homography transformation based on the pinhole model between the image planes, considering the positions of the UAV camera during its flight. Through experimentation, we have configured the UAV to hover mode, allowing us to categorize its motion into two essential types: roll and translation in three directions (for a detailed analysis on this hypothesis, please refer to **Supplementary Figures 5** and **6**). As a result of the UAV's movement, the captured images undergo changes between two moments. These changes can be are described by utilizing the marker center coordinates in this study, as elucidated in **Supplementary Figure 2**. For clarity, we denote the center coordinates for all markers in the first frame of the video as:

$$\boldsymbol{u}_1 = \left[c_{1,x}^A, c_{1,x}^B, c_{1,x}^C\right], \boldsymbol{v}_1 = \left[c_{1,y}^A, c_{1,y}^B, c_{1,y}^C\right] \tag{4.1}$$

Additionally, for the *t*-frame, the marker center coordinates are:

$$\boldsymbol{u}_t = \left[c_{t,x}^A, c_{t,x}^B, c_{t,x}^C\right], \boldsymbol{v}_t = \left[c_{t,y}^A, c_{t,y}^B, c_{t,y}^C\right] \tag{4.2}$$

Those marker center points represent the corresponding points of the initial (first frame) and transformed images, respectively. We describe the marker center points in homogeneous coordinates as $[\boldsymbol{u}_1, \boldsymbol{v}_1, 1]^T$ and $[\boldsymbol{u}_t, \boldsymbol{v}_t, 1]^T$. Next, we employ a plane homography transformation to compensate for the camera motion, which can be expressed as follows:

$$\begin{bmatrix} \boldsymbol{u}_1 \\ \boldsymbol{v}_1 \\ 1 \end{bmatrix} = \begin{bmatrix} s \cdot \cos\theta & -s \cdot \sin\theta & \boldsymbol{d}_x \\ s \cdot \sin\theta & s \cdot \cos\theta & \boldsymbol{d}_y \\ 0 & 0 & 1 \end{bmatrix} \begin{bmatrix} \boldsymbol{u}_t \\ \boldsymbol{v}_t \\ 1 \end{bmatrix} \tag{4.3}$$

The parameter θ is our angle of rotation, while $\boldsymbol{d}_x$ and $\boldsymbol{d}_y$ are the *x*-axis and *y*-axis translations, respectively. The additional term s denotes the isotropic scaling factor. In the equation (4.3), there are four unknown parameters. The two reference markers, Mk-A and Mk-B, provide two sets of non-collinear points (*x*-coordinates and *y*-coordinates) that satisfy the requirement for solving the equation. To simplify the notation, we represent the 3 x 3 transformation matrix as $\boldsymbol{M}$. The compensated marker coordinate can be computed by the following equation:

$$[\boldsymbol{u}'_t, \boldsymbol{v}'_t, 1]^T = \boldsymbol{M}[\boldsymbol{u}_t, \boldsymbol{v}_t, 1]^T \tag{4.4}$$

Through the aforementioned process, the marker images in each frame of the UAV video are aligned with the first frame. Furthermore, the compensation ensures that the pixel distance between the marker center coordinates remains below half of the marker pitch. This crucial step serves as the foundation for phase-based moiré analysis, facilitating the precise retrieval of marker center coordinates and enabling the calculation of displacement values with high accuracy.

Supplementary Note 2: Discussion on the possibilities and limitations of the proposed approach

(i) Possibility

By leveraging the developed method, in which markers are affixed to the bridge surface prior to an initial test, the drone's GPS information can be harnessed to navigate to the same position during subsequent periodic inspections autonomously. This functionality facilitates synchronized video recording with the passing test vehicle and seamless image data transmission to a designated cloud server or remote workstation following the test. The system exhibits a high degree of automation across most measurements, rendering it exceptionally well-suited for inspecting bridges in arduous locations, including rural mountainous areas. As a result, this technology harbors significant potential as an indispensable inspection tool, effectively mitigating the projected scarcity of inspectors in the future.

(ii) Limitation

The accuracy of displacement measurement is linked to the selected marker pitch. In this study, the sampling moiré method, known for its superior accuracy of 1/1000th of the marker pitch, was employed. Therefore, measuring minute displacements surpassing 1/1000th of the marker pitch poses a significant challenge. For instance, with 200 mm pitch markers, the measurement limit stands at 0.2mm displacement, making the analysis of displacements of 0.1 mm or less unfeasible. It is noteworthy that in **Supplementary Figure. 12**, we discussed that the grid pitch of the markers is suggested to be over 10 pixels, which implies the requirement upon the distance between the drone camera and testing bridge. Put them all together, the marker size and image capture distance should be adjusted mutually concerning the demand of field test.

Furthermore, the placement of reference markers on the girders at both the left and right ends of the bridge plays a crucial role in the proposed approach. This approach assumes that the fixed girders remain stationary or that the displacements are negligible. Nevertheless, it must be recognized that the potential for measurement error increases when dealing with entire dynamically moving bridges. Such bridges are not suitable for the proposed approach.

Supplementary Table. 1 Comparison of the state of the art for drone-based displacement measurement method.

Year	Authors (*Ref.#*)	Hardware	Core technique	Evaluation field	Performance and accuracy
2018	Yoon, H., Shin, J. & Spencer, B. F. (*Ref. 27*)	Single camera mounted on drone (marker-free)	1. Optical flow-based tracking key point. 2. Bundle adjustment for **6 DoF** correction. 3. Displacement extraction.	Indoor Lab-scale evaluation with **4.6 m** distance	Achieved a RMSE of **2.14 mm**
2019	Zhang, X., *et al.* (*Ref. 28*)	Camera with tile sensor	Motion compensation by using stationary calibration target. **Tilt sensor** is employed to further refine camera movement estimation.	Indoor evaluation Measured at a **9.75m** distance	Achieved avg. RMSE of **1.44 mm** for in-plane displacement
2021	Perry, B. J. & Guo, Y. (*Ref. 30*)	Optical and infrared (IR) cameras	Optical and IR Bi-branch process. Target tracking and **perspective transformation estimation**. Displacement can be computed by comparing the target and reference target coordinates.	Indoor laboratory setting (**0.75 m** above the structure)	Achieved RMSE of **1.65 mm**
2021	Ribeiro, D., *et al.* (*Ref. 32*)	Drone camera, triaxial accelerometer and external video camera. target marker is used	**Inertial Measuring Unit** (IMU) data is used to cancel drone movement. Vision analysis, including tracking and distortion removal, is applied to generate displacement values.	Outdoor small-scale field test with distance of **4.7~ 5.2 m**	Achieved RMSE of **2.02 mm**
**2023**	Ri, S., *et al.* (***This study***)	Single camera mounted on drone (with 3 markers)	**Phase-based** coordinate estimation. Simply yet effective **4 DoF motion modeling**. Further compensation utilizing geo-structure prior.	**Outdoor** real bridge inspection from range of **35m to 100m**	Achieved RMSE of **0.2 mm** for **35m** **0.8 mm** for **85m**

A comprehensive summary of the current state-of-the-art in drone-based displacement measurement is provided in **Supplementary Table 1**. The survey table indicates that the utilization of drone cameras for structural displacement measurement has become a prominent area of research in recent years, with considerable advancements achieved. However, a significant gap remains between concept-proof demonstration experiments and real-world applications in the previous research. Upon comparison, it is evident that the proposed phase-based drone measurement method outperforms conventional 6 DoF models in terms of accuracy and simplicity.

In stark contrast, our developed method attains a notably enhanced level of precision, achieving an accuracy of 0.2 mm across shooting distances from 35 m and 0.8 mm with the distance extending to 85 m. This discernible contrast emphasizes the effectiveness of our proposed 4 DoF model, which amalgamates phase-based image blurring compensation and displacement measurement techniques. Furthermore, this method has been specifically designed to meet the requirements of practical inspection scenarios, making it a promising approach for real-world applications.
