# Supplementary material for: Drone-based displacement measurement of infrastructures utilizing phase information
Source: Nat Commun. 2024 Jan 9;15:395. doi: 10.1038/s41467-023-44649-2 (PMC10776656; doi:10.1038/s41467-023-44649-2)
Supplement: Supplementary file 3 — Description of Additional Supplementary Files [file 41467_2023_44649_MOESM3_ESM.pdf]

Description of Additional Supplementary Files

File Name: Supplementary Movie 1
Description: Comparison video without and with image stabilization.

File Name: Supplementary Movie 2
Description: Comparison of image blurring compensation for the central marker.
